# Capturing and cultivating the simulated patient/participant (SP) experience: a qualitative study exploring how the perspectives of SPs can inform the co-production of an orientation resource guide

**DOI:** 10.1186/s41077-025-00334-6

**Published:** 2025-03-24

**Authors:** Ellen Davies, Lotte Crawford, Terence Crawford, Renato Musolino, Russell Hutchinson, Lemuel Pelentsov, Michael Gilmour, Adam Montagu

**Affiliations:** 1 Adelaide Health Simulation, Faculty of Health and Medical Sciences, The University of Adelaide, Adelaide, Australia; 2 Faculty of Arts, Business, Law and Economics, The University of Adelaide, Adelaide, Australia; 3 College of Humanities, Arts and Social Sciences, Flinders University, Adelaide, Australia; 4 Clinical and Health Sciences, University of South Australia, Adelaide, Australia

**Keywords:** Co-production, Focus group discussion, Health professions education, Health simulation, Medical education, Qualitative research, Simulated participant, Simulated patient

## Abstract

**Introduction:**

Simulated patients, participants and persons (SPs) are valued members of simulation teams. For people new to working as SPs, there are unique orientation requirements. This project sought to co-produce a resource package with SPs to facilitate orientation to the philosophy and foundations of health simulation, the type of work SPs do and to the structures and environments in which health simulation may be undertaken.

**Aims:**

To explore and describe SPs’ perceptions of their role in health simulation, the things that inform and influence their work, and SP recommendations for orienting new people to health simulation and this type of work.

**Methods:**

Focus group discussions were hosted to construct a narrative with and from people who work as SPs, for the purpose of informing an online resource for new SPs. Data were analysed using Braun and Clark’s Experiential Thematic Analysis methods to address project aims.

**Results:**

Twenty-three SPs participated, contributing their thoughts, experiences and ideas. Data from transcripts were analysed thematically, resulting in three themes, and 11 sub-themes. The broad themes describe (1) The Purpose (why the SP role is valued); (2) The Job (what we do as an SP) and (3) The Craft (how we work as an SP) from the perspective of participants. Specific recommendations for course content were described and integrated into a new non-award, open-access resource for new SPs.

**Conclusion:**

Findings from this study contribute to the ongoing and expanding understanding of the SP role and the perspectives of people who work in the social practice of health simulation.

## Introduction

In health simulation, people who work in the roles of simulated patients, relatives or significant others (i.e. simulated patients/participants/persons (SPs)) [1] come from remarkably varied backgrounds [2–4]. Unlike many others in the health simulation team (for example educators, embedded simulated participants, simulation coordinators, clinicians), SPs do not often have a clinical background to draw from when orienting to simulation modalities of learning and assessment. Consequently, this cohort has varied understandings and interpretations of the purpose and expectations of the work involved [5].

The quality and standard to which orientation and training are delivered for SPs varies depending on the size of the simulation programme and availability of training [5, 6]. Previous studies and commentaries have indicated that orientation and training for people working as SPs need to be enhanced [4–8]. There are principles and standards of best practice which can be followed, as outlined by relevant associations [7, 9, 10], but how this information is packaged and delivered to people who are invariably employed on a casual or voluntary basis requires exploration.

We know from previous research in this area that SPs contribute to simulation programmes in varied ways, and beyond assuming the role of a patient or relative [11]. Amongst other facets, their jobs often include being aware of learners’ progress and performance requirements, providing formal and informal feedback on learner performance, working with interprofessional and interdisciplinary teams, understanding how simulation equipment is safely operated and contributing to the improvement of simulation programmes [3, 11–13].

In our context, people who work as SPs learn about how to work in the SP role *on the job*—that is, they are oriented to simulation environments, techniques, language and concepts whilst employed in the simulation team. Prior to this project, each new SP in the programme was provided with an individualised orientation to the simulation environment and SP techniques for working. As the programme has expanded, the feasibility of this became challenging, and our team wanted to optimise the information and orientation processes that were being provided.

The purpose of this project was to gather and analyse information that could be used to co-produce a package of resources that would be a suitable introduction to the role for new SPs, from the SP perspective. The co-production in this instance would be between currently employed SPs, education specialists and academics, and would blend the perspectives and experiences of SPs with current evidence and principles that underpin health simulation. We believed that providing this type of co-produced, curated resource could enhance the understanding of pedagogy, the quality of SP engagement with students and SP experience as they commence in a new job. The project was undertaken in two parts: first, the exploration of how SPs perceive their role in health simulation, what informs their practice, and how they would recommend new people are oriented to this type of work; and second, the development of the resource package. The focus of this paper describes former part.

Before embarking on the project, we examined our own assumptions, constraints and aspirations that would inevitably contribute to shaping the design of the final resource package. These are outlined here to equip readers with the context in which this project was founded.

First, we acknowledge that SP roles vary significantly, ranging from brief physical examinations (e.g. vital signs assessment) to complex conversations (e.g. about death and dying) with many presentation types in between. Not only do SPs portray these roles, but they also provide feedback to students on their performance—a valued and valid form of feedback [4, 7]. Second, there is a growing number of simulation programmes wanting to employ actors and lay-people as SPs. In many of these programmes, the faculty has little or no experience of the work and subsequently is not well-positioned to provide performance advice to SPs. Third, the nature of the work that SPs undertake necessitates casual contracts, or volunteered time, with reduced opportunities to participate in faculty development activities. Whatever resources are developed should, in principle, be packaged concisely.

### Context

Adelaide Health Simulation (AHS) is a specialist healthcare simulation programme within The University of Adelaide (UoA). AHS works with schools (medicine, nursing, allied health, psychology, health science) to support learning and assessment in simulated environments. Accredited with the Society of Simulation in Healthcare (SSH), AHS has a core team of 18 academic and professional staff and employs tutors and SPs on a casual basis to meet programme needs.

Eighty SPs are employed, with 76 on casual contracts, one employed on a full-time permanent contract to coordinate the SP Program and three full-time, permanently employed simulation technicians who are trained actors and work in the SP role on occasion. The ages of SPs in this programme range from 22 to 84. Thirty-seven identify as female (including one trans-female), 41 identify as male and two identify as non-binary. There is a combination of actor-trained (*n* = 53) and non-actor-trained (*n* = 27) SPs. Australia is a multi-cultural country, and our SP pool reflects some of this diversity. SPs who work at AHS come from several places in the world, including from Croatia, England, Hong Kong, India, Italy, Portugal, Scotland, Serbia and South Sudan. Two SPs are Aboriginal Australian.

The total number of SP casual hours for 2023 was 12,476, with approximately a third of these hours worked for Objective Structured Clinical Examinations (OSCEs) (4523 h). OSCEs were conducted for students of the UoA health disciplines and for external industry clients. For the remainder of the time, SPs are integral to the delivery of experiential learning activities for predominantly undergraduate health professions students. Cases range from clinical skills sessions (e.g. physical examination) to basic ‘health history taking’ scenarios to immersive scenarios where SPs are relied upon to create the social and emotional fidelity of complex cases (for example, scenarios where patients are informed they have a life-limiting illness [4]).

AHS endorses and adheres in principle and practice to the Association of Standardised Patient Educator (ASPE) Standards of Best Practice for working with SPs [9]. An SP coordinator (who is herself a trained actor, SP and co-author of this paper (LC)) works in a full-time capacity to recruit, orient, train, provide feedback to and manage concerns with the SP workforce. This is comprehensive of supporting SPs to provide feedback to simulation participants in debrief conversations. AHS values professional development opportunities for all members of staff and has invested in opportunities for SPs to develop their skills with the State Theatre Company of South Australia, as well as regular informal professional development opportunities (for example, coaching to work in more complex SP scenarios, support to provide feedback to students, training for how to appropriately apply moulage).

### Terms

We are aware of the current academic dialogue relating to the narrow definition the term ‘simulated patient’ conjures [7]. In practice, SPs at AHS play the roles of patients, non-health professional bystanders, relatives, friends and others, but the nomenclature has not yet shifted away from ‘simulated patient’. Therefore, many of the quotes cited in this paper will use this term. Throughout the remainder of the paper, ‘SPs’ refers to simulated patients, simulated participants and the SSH Dictionary term ‘simulated person’ [1].

## Methods

Constructivist, interpretivist and pragmatist paradigms framed the qualitative research approach and lens of the project. Through the lens of social constructivism, researchers can explore how people interact in social contexts, whilst interpretivism allows researchers to explore people’s perceptions of their contexts and experiences [14]. These paradigms are familiar to the investigators as they form a strong part of the philosophy that underpins health simulation in practice. Relevant to this research project, we wanted to view the social context of health simulation from the perspective of SPs and to construct, with this group, a narrative that would benefit novice SPs as they are orientating to the job. These themes have significant overlay with Dewey’s pragmatism, which focuses on people’s experiences and promotes cooperation and empowerment of people to improve their collective situation [15, 16].

The research questions being addressed in this project included: ‘How do SPs perceive their role in health simulation?’; ‘What informs and influences SP ways of working?’ and ‘How would SPs recommend new people are orientated to this type of work?’.

### Participants and recruitment

People were eligible to participate if they were currently employed as an SP. There were no exclusion criteria relating to how long or how frequently people had worked as an SP. We valued the idea of exploring the diversity of experiences of people who were novice and experienced in SP work. An email invitation was sent to all employees on the AHS SP database. The dates and times that were scheduled for focus groups aligned with times where larger cohorts of SPs were working, to optimise recruitment. Participants were offered a $50 Mastercard Voucher at the conclusion of the focus group discussions.

### Data collection

Data were collected via focus group discussions. Focus groups can harness group dynamics to stimulate discussion and the brainstorming of concepts and ideas [17, 18]—a feature that aligned well with the purpose of this study. Participants attended focus groups in person, on campus, for 1 h. Each discussion was facilitated by the lead author and supported by 1 or 2 of the other listed authors (LC, TC, RH, AM). The role of the facilitators was to (1) foster an environment for equitable participation; (2) prompt discussion relevant to the research project with pre-formulated and spontaneous questions; (3) identify moments of divergence in the discussion and (4) probe participants to better understand divergence and convergence of experience and ideas [19]. Facilitators had a brief guide to support the discussion. This guide included four prompts, starting with inviting each member to briefly share their experiences of working as an SP, comprehensive of their length of time working as an SP, reasons for starting this work and types of SP work that they have been involved with. Three questions shaped the remainder of the discussion: What were the challenges when starting to work as an SP?, What would be helpful for people new to this type of work? What formats for providing information and training opportunities would be helpful for people to learn about working as an SP? Discussions were audio-recorded and transcribed verbatim by an external professional.

### Data analysis

Data analysis was guided by Braun and Clark’s description of Experiential Thematic Analysis (TA) [20, 21]. In Experiential TA, participants’ ‘lived experiences, views, perspectives and behaviours’ are explored in six steps of analysis [20]. Descriptions of each stage of analysis are provided in Table 1.

**Table 1:** Description of experiential thematic analysis process

Step	Description
1. Familiarisation	Audio recordings of focus group discussions were heard whilst reading transcripts to confirm accuracy of the transcripts and to commence the familiarisation process. Potential codes and key statements of interest were noted throughout this process.
2. Coding	Coding was formally conducted using NVivo software [22]. Codes were devised to capture interesting and relevant features of the data in concise and brief phrases or descriptive words. Codes can be seen in Fig. 1. This process continued until all relevant data were captured in at least one code.
3. Initial themes	Codes were grouped and re-grouped where it appeared there was shared or complementary meaning, concepts or key ideas. This process was iterative and discussed amongst the group via online and in-person group discussions and shared documents.
4. Reviewing and developing themes	Using the preliminary clusters of codes, themes and sub-themes were developed. Initially, two major themes were identified with multiple sub-themes mostly appearing to be a ‘good-fit’ for these themes. As the review progressed, these themes did not adequately explain the data, and sub-themes were re-distributed amongst three themes which appear to capture all the key concepts and ideas that were identified.
5. Refining, defining and re-naming themes	In re-fining and defining sub-themes and themes, it was identified that different words have different common meanings amongst health and arts focused groups (for example ‘role’: role in a team (health) vs character (performing arts)). Discussions about this led to the styling of the theme names. Sub-theme names are all short quotes from the raw data. These were carefully selected to represent the key features and concepts identified from the collated data.
6. Producing the report	ED led the writing of the report and used qualitative research reporting guidelines to support this process [23, 24]. TC and RM assisted with bridging language differences between the health and performing arts disciplines, and read and re-read transcripts to ensure no key data was absent in the final reporting of themes. All authors participated in reviewing and re-drafting the final report, providing valuable feedback on wording and descriptions of concepts.

**Fig. 1:**
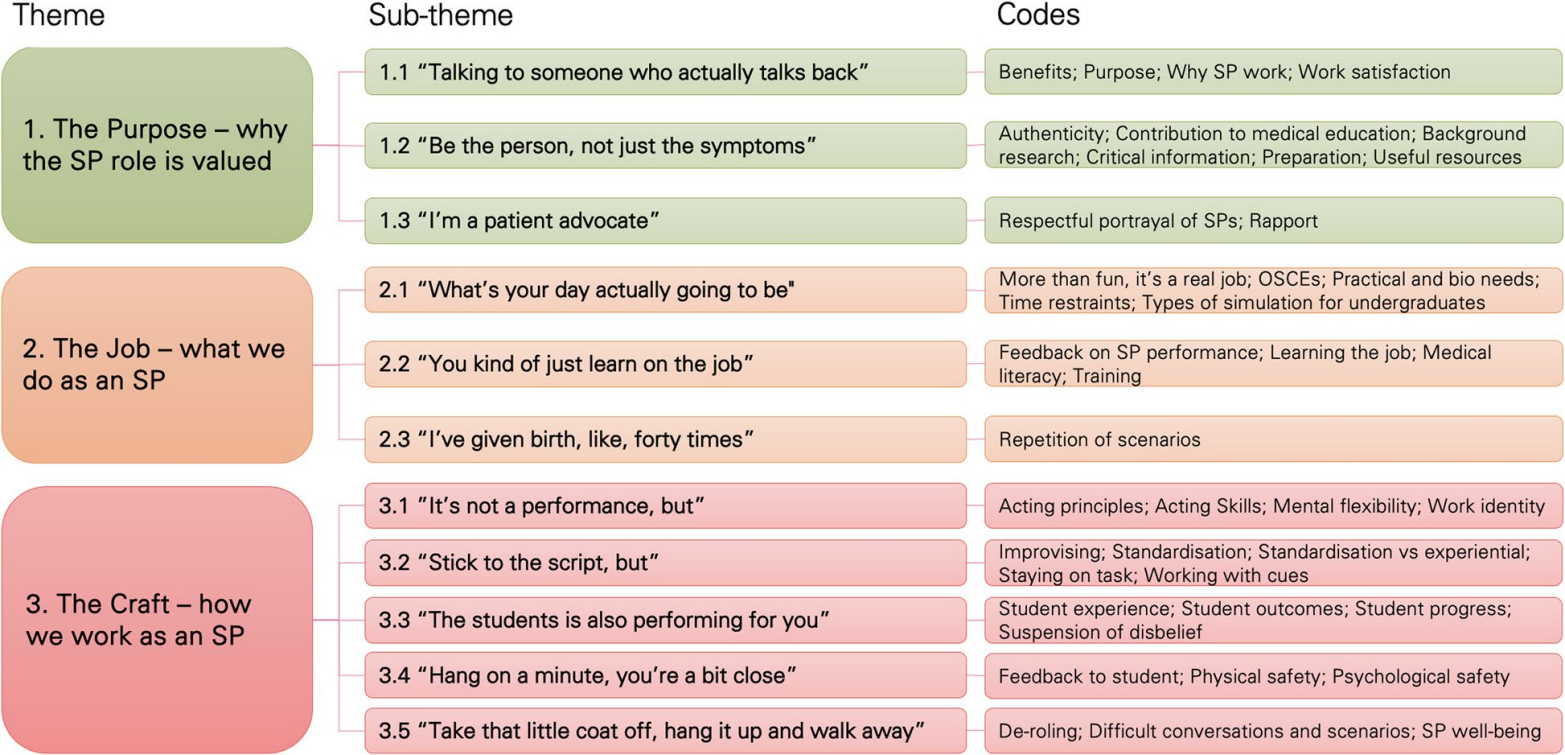
Themes, subthemes and codes]

Throughout analysis, and in consideration of methods used for data collection, results are presented to ‘capture the way in which meaning [was] negotiated and co-produced in the group context’ [18]. Quotations are cited as originating from a focus group (i.e. FG1, FG2, FG3, FG4) and are not attributed to individual participants.

### Data reporting

This paper reports findings from focus group discussions, guided by Braun and Clarke [20], and in accordance with reporting guidelines for qualitative research [22, 23]. The first two research questions are addressed in the three major themes. Findings that address the third research question were directly extracted from transcripts, summarised, categorised and organised into a table.

### Reflexivity

The authors of this paper come from performing arts and clinical health backgrounds, are at various career stages and share common ground in designing and delivering educational programmes in their respective fields. We pragmatically approached the current project, applying our collective knowledge of research methods and resource development to produce a quality, evidence-supported resource for new SPs.

This study adopted the philosophy of collaboration that underpins the Arts and Health Alliance grant scheme that funded the project (gaha.org). The grant required investigators to come from Arts and Health Faculties from at least two of the three South Australian Universities. The investigators come from four faculties (two Arts and two Health faculties) and three Universities (The University of Adelaide (UoA), Flinders University and the University of South Australia).

In keeping with the qualitative approach adopted in this paper [20], the authors met regularly, both in person and virtually to reflect on the processes used throughout the study, and the codes, themes and findings that were identified from focus group discussions. Having authors who have worked as SPs (LC, RH, TC, MG), who have worked with SPs (ED, AM) and who have come from backgrounds outside of simulation (LP, RM) required dialogue relating to the language used to describe concepts, the shared and differing meanings assigned to terms, and the decisions that were made about where and how to include participants’ voices in the final manuscript.

### Ethics

Ethics approval for this project was granted by the lower-risk Human Research Ethics Committee of The University of Adelaide (H-2023–100).

## Results

Four focus groups were hosted in June 2023. The minimum desired number of participants [20] were recruited (total participants = 23) in response to one email invitation. All focus groups were hosted by the lead author and at least one other member of the authorship team. Focus groups were scheduled to align with times SPs were working at AHS—there was a consequent unevenness in group sizes, with two larger groups (Focus Group (FG) 1, *n* = 10; FG3, *n* = 9) and two smaller groups (FG2, *n* = 2; FG4, *n* = 2). Participants ranged in age from 22 to 84 years. They included novice (for one participant, it was their first week) and more experienced SPs (i.e. working as SP for > 10 years). Eleven participants were trained actors. The remaining 12 were not formally trained in the performing arts, but many participate in amateur theatre productions or other performance modalities.

### Findings

Three themes and eleven sub-themes were identified (see Fig. 1 for overview). Themes 1 and 2 provide an overview of how participants perceive their role through the lenses of its purpose and value, and what SPs do in whilst working in this job. Theme 3 explores the ways in which SPs work and the influences that inform this work. Sub-themes are named with short participant quotes and collectively present an overview of the attitudes, perspectives and ideas that were discussed. In keeping with the principles of experiential thematic analysis [3], we wanted participants to identify with the analysed data and, as such, used their words to name each sub-theme.

### Theme 1: The Purpose—why the SP role is valued

The three sub-themes identified in Theme 1 convey participants’ understanding of the intent of SP roles in health simulation scenarios and explores the perceived value of SP work to health professions leaners and healthcare recipients. The benefits of SPs are explored in Sub-theme 1.1 (‘Talking to someone who actually talks’). Exploration and explanations about participants’ attitudes towards their work are described in Sub-themes 1.2 and 1.3 (‘Be[ing] the person, not just the symptoms’ and ‘I’m a patient advocate’).

#### Sub-theme 1.1: ‘Talking to someone who actually talks’

One of the primary benefits participants identified was the opportunity for learners to practice their communication skills with a person who could authentically respond: ‘it’s a good experience…for [students] to be talking to someone who actually talks back rather than…a [manikin] on a bed” (FG3). Further benefits included learner opportunities to develop communication skills ‘to draw out how I'm doing, and what… the condition is’ (FG2), and the preparation learners gained in anticipation of future clinical situations: ‘so that when they go out into the real world they don’t get as overwhelmed’ (FG1). Participants recognised their role in preparing students for professional practice.

The value of SPs went beyond being the recipient of communication. SP work was conceptualised as collaborative story-telling process, with value existing in the collaboration learners buy-in to when participating in simulation scenarios:

I liken it a lot more to Dungeons & Dragons than performing on a stage. Because both of you… know the artifice is there, but in that moment… the student is also performing for you… they know that you’re not actually sick but they’re going to pretend that you are… And so, it’s got a lot more in common with that collaborative storytelling involved in role-playing scenarios… than an actor with an audience. FG1

#### Sub-theme 1.2: ‘Be[ing] the person, not just the symptoms’

Participants confidently described the essence of the SP role: ‘…it’s basically being a real person and responding in the moment’ (FG1). They also articulated their understanding of the benefits of the SP modality when compared to other types of patient replacement in the simulated environment: ‘I mean, you can have all the whiz-bang kind of gadgets and, you know, manikins, etc., but… it’s bringing that real person who you have to communicate with’ (FG1).

Different rationales for having a ‘real’ person to communicate with were identified. SPs’ roles in facilitating technical and behavioural skill development were seen to be particularly beneficial: ‘we try to reproduce the symptoms and be the person, not just the symptoms, because you've got to be a whole person, for… students so that they can negotiate around… aspects of their professional life’ (FG1). Communication challenges that health professionals will invariably face in clinical practice were used to underscore unique benefits of working with SPs:

Not every patient is able to communicate what’s wrong with them… People are inarticulate, people are reluctant…. The student’s got to find a way…to draw out the…the critical moment that they really need to make the diagnosis (FG1).

Participants provided insight into how they ‘become’ the person, without just presenting a selection of symptoms. This was conceptualised as presenting the SP’s ‘truth’. ‘Well, I think anyone who’s been acting for a long time or has trained as an actor is pretty good at just being truthful.. just playing the truth of…of that character …whatever that character’s reality is’ (FG2). Being an SP was described as being more than indicating the traits of the character: ‘when [you] just put actions and facial expressions on top of lines… it just doesn’t…it’s not believable’ (FG2).

In the following illustration, the way that one participant experienced embodying and portraying an SP’s story is described. What is encapsulated in this illustration is the surprise at the depth of emotion they felt as a result of taking on the character, and feeling the emotions that would likely have been experienced by someone who was in the character’s position:

the scenario was a… an Intensive Care situation, and the patient … was in the last stages of Motor Neurone Disease. …And he was my partner, so it was a gay relationship. He had known for many years that this was going to happen, so we were prepared …but what I wasn't prepared for was that he had to die in hospital because the equipment that he needed couldn't be brought home. And we had this plan that… we’d take his bed into the front room, the lounge room, looking through the big windows we have there out over the garden that he built and cared for twenty-five, thirty years, so I was really passionate about that. And because I got so excited imagining and seeing this image, when the doctor told me “No, he can’t be taken home because he needs to stay here for…to be sustained on the equipment”, I just lost it.: I was in floods of tears. If you’re living in the moment with the character these things will just come… naturally (FG2)

This description clearly describes an SP going beyond ‘just’ the script, and ‘being’ the person.

#### Sub-theme 1.3: ‘I’m a patient advocate’.

Advocacy for patients was introduced into the discussion. Participants wanted to be authentic in the SP role, particularly when representing vulnerable people or populations: ‘what I realise now is I want to be the most authentic dementia patient I can’ (FG2). Advocacy was also linked to an intention to not reduce, ridicule or mis-represent real patients: ‘I often get scared of, um, looking like I'm making fun of something, um, which is something I always have to ask a lot of questions around, how to portray it truthfully… because I don’t want to look like I'm mocking someone’ (FG2).

For some, personal encounters with people who have become ill, or interactions with health services increased their understanding of the patient experience:

I’ve learnt now that my partner has been diagnosed with Alzheimer’s disease…it’s pretty easy to deal with at this stage. Later, it’s going to become a hell of a lot of a challenge (FG2).

For others, poor experiences with health professionals lead to an understanding of what specific feedback might be helpful to increase new health professionals’ behavioural skills:

you’ve got a doctor that's sort of like [blah blah blah (quickly)] and… you think “I don’t want to go and see them again”. So, it’s easier to get them when they’re year one or two and say, you know, “Speak a bit slower. Particularly if it’s an older person (FG3).

Participants recognised that simulation with SPs could be challenging, but also that it was done with consideration to everyone’s safety: ‘we do challenge them, and that…you know, sometimes they are more difficult situations that they’ve had to go through, in a safe space’ (FG4). Encouragingly, participants witnessed the benefits to learners of participating in simulated scenarios: ‘do you know… how many times I’ve heard students come back and go “Do you know what? I had that same scenario happen to me in the hospital and it all came flooding back to me and I knew what to do’ (FG4). Participants were enthusiastic about their involvement in learners’ professional development and saw this as having a direct impact on the safety and well-being of future patients.

### Theme 2: The Job—what we do as an SP

Theme 2 describes many of the practical aspects of working as an SP. It describes work expectations in sub-theme 2.1 (“What’s your day actually going to be”); describes participants’ experiences of learning the job in sub-theme 2.2 (“You kind of just learn on the job”) and the sometimes repetitive nature of the SP work in sub-theme 2.3 (“I’ve given birth, like, forty times”).

#### Sub-theme 2.1: ‘What’s your day going to actually be’

Participants agreed that the role of the SP is ‘more than fun, it’s a real job’ (FG3). They went further to explain: ‘people go “Oh, your job’s so easy! I wish I could lie in a bed all day!”, but then that’s not what it always is, you also need to be able to do all these other things that require a lot of acting’ (FG2). And whilst some of the time, lying in bed waiting to be examined by students was a reality, it was not a defining feature of the job.

Participants noted how the different types of simulation activities shaped their day. Broadly, there were obvious differences between experiential learning days (where students are practicing a skill and being provided expert feedback), and days where objective structured clinical exams (OSCEs) were being held (where student competence is being formally assessed). OSCE days were particularly recognised for specific rules, for example ‘… you can’t go outside’ (FG3).

In terms of working on OSCE days, due to the nature of these often high-stakes assessments, participants noted the principle of standardisation: ‘…you don’t give help to anyone, like, in an OSCE; you give it the same to every single person’ (FG4). They were also aware of the student experience, acknowledging their limited time (‘it’s an artificial structure, so you…have to be clear about that. It’s eight minutes…’ (FG3)), and acknowledging a desire to give the students the time they need to perform within the time limitation (‘I'm always conscious of not taking up too much of their time, so staying out of the way a little bit’ (FG3)). A further point of difference was the type of answers students might seek from an SP in an OSCE setting, as opposed to an experiential learning context: ‘the timelines are so tight that they really don’t have a lot to get into that background story’ (FG3).

For days when working in simulations designed for learning, participants’ various experiences were noted. Some days involve basic examinations and history-taking conversations:

I would say my most common roles have been…well, one just being for examinations, physical examinations, so that’s no character, just kind of being a body there for young students to practice their technical skills and then basic consent and communication skills (FG1).

Some involve working as an SP in heightened emotional states and others involve days where the SP has physical challenges that need to be emulated. There is substantial variation in what SPs will do from day to day. These variations stem from the requirements of the programme that is being held and the purpose of the simulation (i.e. experiential vs. assessment).

#### Sub-theme 2.2: ‘You kind of just learn on the job’

Participants reflected on how they learned to work as SPs. This included developing some medical literacy, learning how to interpret information provided for scenario preparation, and navigating inconsistencies arising from working with different tutors and disciplines.

Medical jargon was commonly encountered in SP briefing paperwork: ‘A lot of the time when we get sent a brief… that will all be in medical jargon’ (FG2). As an example, the term ‘anorexia’ was used in reference to a symptom, and not with its more commonly used meaning relating to a diagnosis:

in the brief it said “Patient has some anorexia”, and I was like “Some…? Some anorexia?”… so I was like “Okay, so she’s…maybe she’s like newly anorexic?”… But then, when I got there and I asked the examiner, they just said “Oh, that just means that she’s not hungry at the moment (FG2).

Instructions for how to respond to learners also contained unfamiliar medical terminology. For example: ‘when they palpate the medial joint line’ (FG1). This was challenging for SPs who were not familiar with terms, and who also recognised the importance of not being overly familiar with medical terms when being assessed by learners who need to communicate in ‘layman’s’ terms.

you don’t necessarily know what “palpating the stomach” means, or “percussing” or “auscultating” ... And obviously you don’t want to be *au fait* with all the terminology because the students should really fill you in if you’re going “I don’t know what you mean" (FG1).

In times when medical terms needed to be understood to learn a role, almost all participants acknowledged using an internet search engine: ‘I’ll Google it’ (FG3).

Learning *on the job* involved becoming familiar with how to interpret the scripts or other information provided for performing in various SP roles: ‘You’ve got to know what’s important in all the information they give you. And a lot of the information you’re given is … unnecessary…you can spend a lot of time learning [stuff] that you’ll never use’ (FG1). Discussing details was helpful for gaining clarity: ‘going over the character in general with each other. Um, usually talking with the instructors or supervisors as well.’ (FG3). For people new to the SP role, learning about the physical nature of the role was also described: ‘at times you will have to expose, um, to the bra or a pair of shorts, and the students might have to touch certain areas of your body, but if you’re at any time uncomfortable you just speak up’ (FG2).

Participants noted that there are often inconsistences to navigate and a consequential requirement to adjust certain aspects of their SP portrayal:

when you have the tutorials, and you have two different doctors coming in from two different disciplines, one in the morning one in the afternoon, you might have one who’s a GP and one who’s an Emergency Medicine person, and they’d have totally different outlooks on how that [presenting complaint] should be dealt with (FG3).

To manage discrepancies one participant offered the following:

My advice on the first day would be make use of the…the examiner or the tutor or whoever’s the, um, actual medical professional in the room… Just ask “Is that…was that right? What would you like me to do differently?” (FG1).

#### Sub-theme 2.3: ‘I’ve given birth, like, forty times’

Summarising the SP job, participants recognised that ‘there’s a lot of repetition’ (FG1). One participant who works in the performing arts summarised the scale of repetition, saying ‘we talk [in the arts] about having to do eight shows a week, well we [as SPs] have to do thirty shows a day!’ (FG4).

Skills important to performing artists were valued as important in SP jobs: ‘…actors have the ability… to remember, but also reproduce, and…that’s probably one of the most important things, you need to be able to reproduce the same way every single time’ (FG4). The requirement of this repetition for the SP was the re-setting for the next learner group:

depending on the scenario… you have to build up to a certain point. But then… I’ve got to check back to zero again, erase what’s just happened, remove tears, whatever, so that it…yeah, again, the journey people are getting is similar (FG1).

Working the same case repeatedly came with some considerations. These related to physical touch (‘… [you] have to physically know what you’re capable of. Because, you do physical exams, there are going to be… students touching you all day’ (FG1)); to energy expenditure (‘it’s sort of like calibrating your energy levels, knowing that… you do it again and again and again (FG1)); and to the potential emotional toll (‘the performance of someone who’s upset becomes, um, something that’s difficult to replicate all the time’ (FG4)).

### Theme 3: The Craft—how we work as an SP

Theme 3 explores the skills and attitudes that participants believed are required to work in SP roles. These include the transferred skills learned from acting training, as well as from life experiences. Five sub-themes are detailed, with a focus on ‘the craft’ of working as an SP.

#### Sub-theme 3.1: ‘It’s not a performance, but’

Participants who had trained and worked in the performing arts recognised the benefits of their training. The point of differentiation between traditional acting roles and SP work was identified by the focus on the learner (‘it’s for the students, it’s not for you to progress your acting career’ (FG4)) and not the performance of the SP (‘I’m performing a role…but I’m not doing it for the glory of my acting’ (FG1)).

Participants expressed great satisfaction in SP work from a performance point of view: ‘it’s just so satisfying to…to realise how broadly you can spread your skills’ (FG2). Pride was taken in identifying with the acting profession: ‘it makes me feel better to say “What I do is acting; I'm an actor”’ (FG2).

It was identified that SP work is not easy—participants described the craft required to take a description from the page to give life to the SP: ‘people think that anyone can do it… there are some scenarios that most people could do… but then… you also need to be able to do all these other things that require a lot of acting [skill]’ (FG2). This skill and this work require more than something mechanical. It was described as ‘…tuning into what it is to be human’ (FG1).

Participants felt that the work they were doing as an SP was not always favourably perceived by others outside of the simulation environment: ‘I think it’s important, as well, saying…like, talking about the skills that we bring to the job. Because I think there’s a bit of a stigma around this work, that people think it’s really easy’ (FG2).

In becoming attuned to the SP role, participants reflected on principles that would be helpful for working in SP roles for those without acting training:

what you’ve got to do with non-actors, the thing you’ve got to convince them of is that the thing they must not do is act…. You know, like that’s…that’s the thing that all of us when we start out have to…have to learn… That what it looks like actors are doing is not actually what they’re doing (FG4)

And also reflected on what is helpful to transition from performance arts to SP work:

I reckon the most helpful thing for me…was actually doing a mock one with [the SP coordinator]... Because whilst I’d done a million role plays elsewhere, the OSCE stuff is so specific. So, I could actually see the difference between open-ended roleplay and specific outcome roleplay (FG3)

Whilst not a traditional performance, elements from the performing arts are necessarily incorporated into SP work.

#### Sub-theme 3.2: ‘Stick to the script, but’

Not all learners will enter the simulation in the same way. There will be variations in the type of questions asked and the behavioural skills of the different learners and learner groups. For this reason, it was recognised that, as an SP, there is a requirement to: ‘stick to the script so it’s standard, but tailor it to what you’re being given in that situation’ (FG1).

In the SP experience, there is a reasonably standard approach learners use to seek demographic information. (‘The students get into a way, particularly year one and twos, to say “What’s your name? What’s your date of birth? What’s your address?”, so, you need to have that in the back of your mind so you can quickly do that.’ (FG3)) In efforts to build rapport, learners may ask questions for which the SP was not provided information (Examples include: ‘…you’ve got a dog, “Oh, what’s your dog’s name?’”(FG3) and ‘your script might say that you’re a sheep farmer over on the Eyre Peninsula’, and they may say ‘Oh, do you know so and so from, you know, Port Lincoln?’ (FG3)).

At times, improvising isn’t problematic (e.g. making up the name of a pet). However, participants recognised the need ‘not [to] give away something that might… take [learners] on a different path’ (FG4). ‘Sticking to the script’ entails ensuring that critical information is presented at the time it is called for in a scenario. It also entails

understand[ing] that the time they’ve got, they’re going to concentrate on the medical condition (FG3). Distractions from improvisation that detract from the case at hand were recognised as problematic for both learning and assessment events.

The timing for providing critical information to learners was discussed in terms of ‘working with cues’. ‘There are sometimes key markers within our script that you hit if you’re given the right information or communicated well, you know, with the students. And if you don’t get those, you don’t offer it’ (FG1). Participants explained that ‘if the student… gives the right signals and clues we then respond accordingly’ (FG1). Participants found script instructions that advised of when not to provide information valuable (for example: ‘Don’t tell them this unless asked’ (FG3)).

Flexibility and capacity to improvise are not only required when learners ask questions that were not anticipated. Adaptability is also required when sessions were changed by tutors. Participants recognised that in working as an SP, they need to ‘think on your feet. You are sometimes thrown things that are unexpected, uh, “We’ve suddenly changed the scenario, so we’re going to include this”. So, you have to be prepared to be flexible’ (FG4).

#### Sub-theme 3.3: ‘The student is also performing for you’

The idea that the student is performing for the SP turned participant discussions to student performance, experience, outcomes and the suspension of disbelief required for simulation to be optimised. Learner performance as a health professional was understood to include their behavioural skills: ‘their mannerisms are very important—the way that they portray their-self to you’ (FG3), and their clinical acumen. Practicing performance was seen as an important for bridging the student-to-professional gap: ‘if they do it here then it will be so much easier when they go out into the workforce. So, I think it’s really important to get that practice’ (FG3).

At times, SPs felt they needed to support learners though emotionally challenging moments of their performance (‘I think that that’s a very important part of being an SP, is to make the…make the student feel like they’re at ease and they’re actually doing the right thing’ (FG3)). One participant offered the following example:

In second year they do sexual education. You know they're doing sexual education, they’ve got me as a seventy-five year old… “…are you still having your periods?”… so, what I have done when they say is say “Hang on a minute, I can’t think back that long”… you know, just to make it a little bit lighter, because they’re so embarrassed and I don’t want to embarrass them more. (FG3)

Participant performance anxiety was perceived positively: ‘it’s also kind of nice, in a way…to see them get flustered. Because, to me, that shows that they’re suspending their disbelief and they’re taking it seriously’ (FG1).

Learners do not always immediately buy-in to the SP. At times, participants felt they needed to facilitate learners’ suspension of disbelief, by bringing their attention back to the experience of the patient they were embodying: ‘I’ve definitely been in scenarios before where they’ve laughed. Um, and a big one is, halfway through, they’ll say “Oh, you’re such a good actor… You’re doing really good acting”. And…I’m like “What do you mean? I’m not acting. What do you mean I’m a good…”, like, “What?”’. (FG2). In instances where learners were giggling or laughing, participants remained in character to get scenarios back on track:

What the hell are you finding so funny? I’m talking…I’m talking about how, um, I think I’m,…my…my wife is going to walk out on me or “I’ve got a divorce pending and I'm gonna lose access to my kids, and you’re laughing?”, that sort of thing” (FG2).

Participants saw their role in scenarios as supporting the performance of learners, and facilitating an environment that would replicate the types of stress and responses that would be experienced in future environments.

#### Sub-theme 3.4: ‘Hang on a minute, you’re a bit close’

SPs will often provide feedback to learners at the conclusion of a scenario. Participants described the instructions they had received for providing feedback: ‘Always give feedback on how you felt as the patient’ (FG2). As an example of this feedback, a participant offered the following:

We’re about dealing with their communication. “How did you deal with me as a patient? …Look, you maintained eye contact with me, you actually brought your chair closer to me… that showed you’re actually involved.. If you move your chair away, well what’s wrong? Is my breath bad, or is there something wrong? So, you moved in close and you've maintained eye contact and you responded to what I said… …you didn’t just ignore me, you responded to what I said, showed me you were listening”… (FG3)

Importantly, participants attempted to provide feedback about the positive aspects of an interaction: ‘I usually try and find one really good thing the student did to keep in the back of my head for feedback. So, if that’s all they get in amongst perhaps “Oh, you maybe could’ve done this or that, you know, there’s one good thing that's a highlight. And there usually is, even if it’s a small, small highlight”’ (FG3).

One noted benefit of the SP experiencing an interaction with a learner is the ability to provide feedback on uncomfortable moments: ‘all those things that you don’t get from the manikin; going, um, “You know, when you lean over me you’re really pushing on my ribs there”’ (FG 1). Another example included: ‘Well, okay, I felt that you actually dismissed me on that point. I didn’t feel you were including me in the conversation’ (FG3). Participants recognised the importance of considering the desired learning outcomes of sessions when formulating feedback: ‘you’ve got to put things in a way that can be helpful. And that are…that are in the…a sensible context of where they’re at and what the aims of the exercise are’ (FG3).

With the increasing use of telehealth, simulations using online platforms have also increased. These brought different communication challenges and topics for SP feedback:

I have been doing Zoom ones as well. That’s quite different… because you can’t get as much physicality on Zoom… I was asked to give feedback and I said “I rather like the top of your head and the way that you've got your hair” because that was all I could see. Because they’re down like this (gestures head looking at desk, not at camera, and I’m…and all I can see is [their hair] (FG4).

Providing feedback was recognised as integral to the SP role and an opportunity to contribute to the improved behavioural skills of learners.

#### Sub-theme 3.5: ‘Take that little coat off, hang it up, and walk away’

This final sub-theme relates to ‘de-rolling’. In the words of a participant, this is: ‘how to create that distance between yourself and the character you’re playing…(FG1). This skill was further explained:

actors learn how to separate themselves, because they know that they…they inhabit the character for a particular period of time, and then they take that little coat off—depending on what your approach to acting is—and they hang it up and they walk away, and then they can be themselves. All the problems that that character has lives in the jacket. And when they need to put it on again they can inhabit that role again…. it’s kind of…you’re really separating yourself from…from the patient, from the client. It’s…these things are happening to that person… (FG4).

Participants acknowledged that: ‘this job isn’t for everyone. And…some people, I think, would benefit from knowing exactly how this works before getting into the job’ (FG2). The ability to disconnect from the SP role was identified as a necessary part of the craft of SP work. Participants noted that:

there is a satisfaction you get when, um, you know, like I did at the other day at the OSCEs a kind of, um, breaking bad news scenario... I think I worked out twenty-eight times… but it’s not actually taxing because I’m not drawing on any personal thing… for me, there’s this well. I can tap into the well, and then afterwards it’s fine (FG1).

When distancing from a ‘bad day’ or challenging SP role, some of the usual techniques to de-role may not work. In these cases, participants recommended talking with other SPs: ‘being able to talk to your other SPs. I’ve had this experience, um, like, we’re all on the same boat and we all have very similar experiences’ (FG4). The role of the Program Support Officer or Coordinator was acknowledged as also important on the occasions where SP cases were more challenging and difficult to distance from:

I just did mandatory reporting for, like, Bachelor of Nursing and, um, yeah, like kudos to [Program Support Officer] who had sent me an email prior and said “Look, you know, let me know if you're not okay. Like, this is the case”, because I said “Yeah, I'm available”, and she was like “This is the case. Let me know if you’re not comfortable with this” (FG1).

Participant recommendations for the resource package.

Participants had numerous suggestions for what information could and should be contained in an online package of resources for novice SPs. These recommendations were extracted from the focus group transcripts, summarised and are categorised in Table 2.

**Table 2:** Recommendations for resource package content

Category	Sub-category	Participant recommendation
**Information to provide**	Key terms	A glossary of commonly used medical and simulation-specific terminology
	Orientation to simulation environment and colleagues	Information about simulation context and equipment
		Information about tutors and their expectations for working in simulation
		Safety considerations when working in a simulation environment with simulation equipment
		Explanations of simulated equipment (for example simulated intravenous cannulas), and how these are applied in a simulated setting
	Key information for what to expect when working as an SP	There is time spent waiting before and after students
		There can be a lot of repetition of the same cases, and SP performance needs to be regulated so that the learner experience is consistent
		There are different expectations and ways of working for different types of simulation (e.g. experiential vs assessment)
		Physical examinations will involve learners touching and examining SP’s bodies—what to expect, what’s acceptable and what’s not
		Expectations for cleaning/tidying up at the end of a shift
	Feedback to learners	Written guide and instructions for how to provide feedback to learners
		Examples of feedback that can be provided to learners
**Format for provision of information**	Audio-visual aids to understand the job	Videos to explain the different types of simulation
		Videos that provide demonstrations of different physical examinations
		Videos of typical case presentations
	External sources to be linked	Information about de-roling
		Information about intimacy co-ordination and consent in theatre/ acting contexts

## Discussion

SPs are a valuable and valued part of health simulation teams [9, 24–26], but whilst other members of simulation teams (particularly medically orientated professionals) have very similar pathways towards the simulation team, SPs enter from a wide variety of work and study histories. There are advantages to not sharing the same degree of medical literacy or technical knowledge of content that is being explored by learners however SPs do require an orientation to the premise of simulation and to the job that they will be undertaking.

In this study, 23 SPs participated in focus groups to explore and describe their perceptions of their role in health simulation, the things that inform and influence their work and their recommendations for orienting new SPs. From these discussions, three themes were identified: ‘The Purpose’, ‘The Job’ and ‘The Craft’. These themes collectively provide a detailed overview from the SP perspective: why the SP role is valued, what SPs do, and how SPs work. These themes capture insights of what it is to be an SP, and what it means to work with learners in a shared, social, learning environment.

Dieckmann et al. describe the socially complex endeavours of health simulation and frame simulation as a social practice [27]. This social practice is goal-oriented and is concerned with the human and social experience of learning about how to be a health professional [28]. Importantly to this study, the social practice lens highlights that all people who are involved in the design and delivery of simulation will have influence on the experience [28]; this includes SPs, who have a function in fashioning the meaning that is made in the social interactions of simulated scenarios.

Described in the findings of this paper are considerations of the social practice of simulation and the functional role of SPs in this social practice. This extends to the artifice of circumstance (simulated scenario) and realism of interaction (genuine clinical conversation). The purpose and role of SPs in health simulation are examined with consideration given to the purpose, day-to-day work and the craft of working in the health simulation context. Participants reflected and recognised the worth of the human-ness of SPs, and as in previous research with this population, described their role as members of the educational team [4, 7, 10]. As members of the educational team, the function of the SP job was described in ways much akin to those in Pritchard et al.’s 2020 ‘It’s not an acting job…’ paper [10]. And indeed, there is much alignment between findings in this paper and in others where SP experiences have been examined [4, 10, 29, 30].

What was particularly interesting was the role SPs felt they had in advocating for patients— the desire to improve learner engagement in person-centred care for the benefit of future patients. This is not a new finding [4, 25, 31], but one worth emphasising as a significant motivator for people who work as SPs. Not only are SPs working with simulation teams to develop health professionals, but they are also actively involved in improving patient outcomes.

Findings underscore that working in simulation environments is different to other types of work, and that orientation to the SP job requires more than learning specific roles or feedback conventions. Table 2 provides pragmatic suggestions for orientating new SPs to the principles, premise, modalities and purpose of simulation. Participant recommendations were considered throughout the co-production of an open-access online resource package for novice SPs (https://the-university-of-adelaide-2657.reach360.com/share/course/9712267e-739d-4426-a758-621cff590bb8). These resources are structured to meet the project aim of providing information that will assist new SPs to orient themselves to the foundational principles of simulation and to the type of roles and work they may be involved with in an SP programme.

### Limitations

This paper included participants who predominantly work in the same simulation context. In this context, the dominant culture (white-Australian) permeates most facets of health profession education. Whilst the data and the resulting resources may be relevant to many in this culture, they are not representative of diverse cultural and environmental practices, beliefs and ways of being.

Due to the nature of the majority of work at AHS, much of the focus of this paper relates to the fields of medical and nursing education. Whilst content may be relevant to other health professions disciplines, there are likely gaps in the subsequent resource and in findings of this paper that would not be representative or relevant beyond this context.

The authors of this paper have their own experiences of working as or with SPs and of exploring other people’s narratives of the SP role. These will have influenced the way in which the data were collected, analysed and interpreted. Open dialogue between authors was consistent and regular and used as a strategy to reduce bias. Despite this, the lenses through which the data have been analysed are subject to biases and experiences as described in the introduction and methods sections of this paper.

## Conclusion

SPs are an integral part of the teaching team but rarely come from health backgrounds. They often do not enter the job with the same mental models, medical literacy or acculturation to health professions practice as learners or educators. Faculty development is often designed for members of simulation teams who have this baseline knowledge and experience, but what may be needed for novice SPs is a more comprehensive contextual orientation. This study worked with SPs to consider how they perceive their role in health simulation, what informs their practice and how they would recommend new people are orientated to this type or work. Delivered from the SP perspective, a new open-access resource was co-developed with members of the research team, informed by relevant published literature and, at its core, SP perspectives, experiences and expertise.

## Data Availability

Data is provided within the manuscript.
